# Dietary Trehalose as a Bioactive Nutrient

**DOI:** 10.3390/nu15061393

**Published:** 2023-03-14

**Authors:** Anqi Chen, Patrick A. Gibney

**Affiliations:** 1 Department of Food Science, Cornell University, Ithaca, NY 14853, USA; 2 Science Center for Future Foods, Jiangnan University, Wuxi 214122, China

**Keywords:** trehalose, *Clostridioides difficile*, diabetes, gut microbiome, sweeteners

## Abstract

Trehalose is a naturally occurring, non-reducing disaccharide comprising two covalently-linked glucose molecules. It possesses unique physiochemical properties, which account for multiple biological roles in a variety of prokaryotic and eukaryotic organisms. In the past few decades, intensive research on trehalose has uncovered its functions, and extended its uses as a sweetener and stabilizer in the food, medical, pharmaceutical, and cosmetic industries. Further, increased dietary trehalose consumption has sparked research on how trehalose affects the gut microbiome. In addition to its role as a dietary sugar, trehalose has gained attention for its ability to modulate glucose homeostasis, and potentially as a therapeutic agent for diabetes. This review discusses the bioactive effects of dietary trehalose, highlighting its promise in future industrial and scientific contributions.

## 1. Introduction

**Figure 1:**
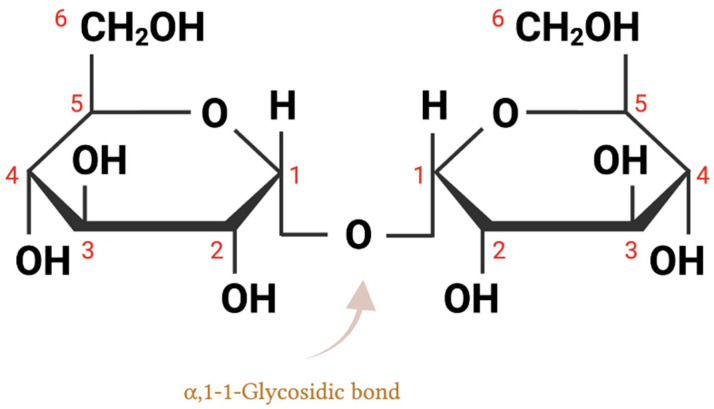
Structural illustration of trehalose, a disaccharide formed by combining two glucose molecules with an α-1,1 glycosidic linkage (α-D-glucopyranosyl-1,1-α-D-glucopyranoside).

Trehalose, a naturally occurring, non-reducing disaccharide, comprises two glucopyranosyl units connected via an α, α−1, 1-glycosidic bond, as shown in Figure 1 [1]. It is widely distributed in many organisms, including bacteria, fungi, insects, plants, and some invertebrate animals [2]. Trehalose contains a non-reducing end hydroxyl group, rendering it a stable molecule not subject to glycation reactions [3]. The structure of trehalose also makes it a molecule highly resistant to acid hydrolysis, as well as a dehydration or cryo-protectant in a variety of microorganisms, plants, and animals [4,5,6].

In recent decades, trehalose has sparked growing interest in a variety of industrial applications [3,7]. This can be attributed to the growing body of research demonstrating the bioprotective properties of trehalose, as well as its absence of toxicity [8]. The introduction of an efficient biocatalytic technology for its synthesis in 1995, enzymatic conversion of starches into trehalose, has also contributed to its remarkable expansion in industrial use over the last two decades [9]. Its distinctive properties that bring about stabilization and sweetness have allowed its various applications as a packaging material, desiccant, drug excipient, and preservative in the food industry [10]. Furthermore, the ability of trehalose to protect a broad array of biological materials, such as DNA, proteins, cell lines, and tissues, has opened up possibilities in the pharmaceutical and biotechnology industries [11]. Trehalose is also gaining attention as a potential human therapeutic, as it has been proposed to mitigate the disease burden in models of neurodegenerative and cardiometabolic diseases [12]. It has also been reported to have various biological effects, such as suppression of bone resorption and inflammation, induction of autophagy, and alleviation of Huntington’s disease [13,14]. However, the precise mechanism or mechanisms underlying these observations remain unclear, and may be complicated by the enzymatic conversion of trehalose into glucose in the human digestive system by either human-encoded or microbially-encoded trehalase enzymes. This leads to open questions regarding how much free trehalose is accessible to diseased tissues, if and how trehalose enters human cells to elicit a physiological response such as induction of autophagy, and whether these or other trehalose-related effects on human physiology are indirect results of the microbial metabolism of trehalose by human microflora [14]. Further research into the role of trehalose and its effect on these disease states is warranted, especially with the appropriate control experiments and validation in a clinical setting.

**Figure 2:**
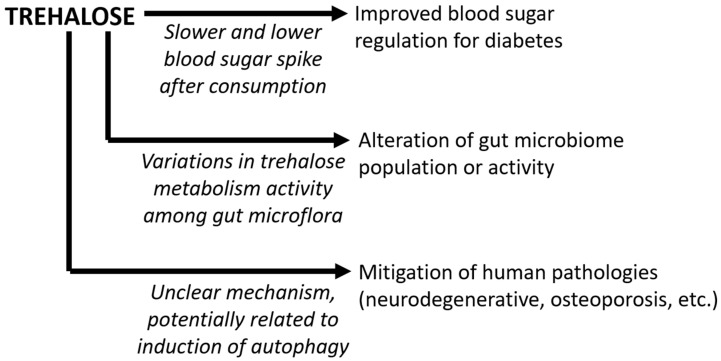
Bioactive roles of trehalose as a dietary nutrient. The three main bioactive roles of trehalose discussed in this manuscript are indicated, along with arrows to indicate the proposed mechanisms of action (text in italics).

Taken together, as a dietary sugar, trehalose has been increasingly consumed over recent years, especially since technological progress has enabled a reduction in its cost. As a bioactive nutrient, studies have proposed that trehalose is a potential tool for regulating blood sugar levels and alleviating diabetes symptoms. Additionally, recent work has focused on the effects of trehalose in the human gut microbiome modulation. The goal of this mini review is to discuss trehalose as a bioactive nutrient, including how trehalose affects the human metabolism and gut microflora populations (Figure 2).

## 2. Trehalose Chemical Structure and Biochemical Properties

Trehalose is composed of two glucose molecules connected by an α, α − 1,1-glycosidic bond (Figure 1). It is believed that α-,α-trehalose is the only form of trehalose that can be isolated from, and biosynthesized in, nature, due to its low conformation energy compared to the other two isomers, α, β-trehalose, and β, β-trehalose [2]. No living organism has been found to contain either α, β-trehalose or β, β-trehalose; however, both can be created through the Koenigs–Knorr reaction [15].

As one of the least chemically reactive sugars in nature, trehalose is non-reducing because the two glucose molecules are linked together by an O-glycosidic bond at the anomeric carbons of the glucopyranose rings [16]. Trehalose does not possess any free aldehyde groups that could take part in reduction processes, such as the Maillard reaction, since both of its anomeric carbons are involved in the disaccharide bond. Its glycosidic bond energy is less than 1 kcal/mol, making it exceptionally stable [17]. In comparison, sucrose, another commonly found nonreducing disaccharide, has a high glycosidic bond energy of 27 kcal/mol [18]. Due to its unique structure, trehalose only breaks down into two reducing monosaccharides under extreme hydrolysis conditions, or in the presence of enzymes, while sucrose quickly decomposes when exposed to reactive amino groups.

Trehalose stands out from other disaccharides due to its non-reducing nature and low glycosidic bond energy, as well as its exceptionally high glass transition temperature [7]. At its glass transition temperature (T_g_), 110–120 °C, trehalose shifts from a solid to a viscous or rubbery state, which can be used as a reference point to explain its stabilizing properties across the solid, glass, and liquid phases [19]. The widespread use of both sucrose and trehalose as a stabilizing agent for a variety of storage purposes is well-known, with disaccharides in general offering stabilizing effects for both cryo-storage and anhydrobiotic preservation (e.g., freeze-drying) [20]. However, trehalose proves to be more advantageous than sucrose in a variety of biological preservation applications, such as protection from heat shock and lyophilization [21]. The high glass transition temperature of trehalose makes it an attractive option for the high-sucrose foods industry to consider for amorphous sucrose–trehalose mixtures [22]. If trehalose is able to effectively reduce the rate of sucrose crystallization in typical storage environments that surpass T_g_, then product shelf life can be prolonged.

## 3. Trehalose as an Increasingly Consumed Dietary Sugar

Before 1995 in Japan, and before the turn of the century in the U.S. and Europe, the vast majority of consumed trehalose was derived from natural sources [23]. It is found in mushrooms, yeast, honey, beans, seaweeds, and shellfish, though the dietary contribution of trehalose from the consumption of all these foods combined is low, compared to added sugars in processed foods [2,24]. The total trehalose consumption in the U.S. was estimated to be at approximately 21 g (per person per year), with natural sources contributing less than 0.3 g [23]. In the mid-1990s, a revolutionary enzymatic technology, based on the liberation of trehalose from starch, was developed in Japan for the industrial scale manufacture of trehalose, resulting in a steep decline in its unit price from USD 700/kg to USD 5–6/kg [7]. Trehalose has been incorporated into various food items, including baked goods, rice and pasta, breakfast cereals, dried fruits, processed vegetables, dairy products, chewing gum, seafood, and ice cream [25]. Confectionery items make up a substantial portion of the total use of trehalose, which has been incorporated into more than 8000 different types of food [3]. The growing use of trehalose in confectionary products can be attributed to its less intense sweetness and longer persistence of sweetness [26]. Due to its growing applications, the average daily intake of trehalose for consumers of all ages in 2019, which was reported by a Generally Recognized As Safe (GRAS) notice, had increased to 42.37 g (per person per day), doubling the amount from two decades ago [27].

While human cells do not encode enzymes to synthesize trehalose, trehalose ingested from food can be hydrolyzed into two glucose molecules in the small intestine by the trehalase enzyme, an enzyme that specifically acts on trehalose [28]. Trehalase activity has also been discovered in kidney, liver, and peripheral lymphocytes, with the proximal and mid-small intestine having the highest concentration, and the distal ileum having the lowest [25]. The glucose molecules released from trehalose can then be actively absorbed and metabolized by intestinal mucosal cells through the glucose transporter 1 (*SGLT1*) [29].

In the human digestive tract, apart from host-produced trehalase, trehalose is also metabolized by microbial-produced trehalases [30]. Microbes can use trehalase enzymes to break down trehalose into glucose, which can then be used in glycolysis. Many intestinal bacteria produce trehalase enzymes, including *E. coli*, *C. difficile*, *Blautia* spp., and *Bacillus* spp. [31,32]. *Bacillus* species use trehalose as a carbon and energy source during exponential growth, while *E. coli* utilizes trehalose as a carbon source through an osmolarity-dependent process [33,34].

Although human consumption and use of trehalose have risen significantly, there have been no reported toxic side effects, aside from rare cases of malabsorption due to trehalose deficiency [25]. Trehalose deficiency is a metabolic condition in which the body lacks functional trehalase enzymes and is not able to convert trehalose into glucose [35]. Affected individuals suffer from abdominal discomfort, vomiting, and diarrhea after eating foods containing trehalose. It is not a common occurrence, yet, at least 8% of Greenland’s population is known to have trehalase deficiency as a result of autosomal dominant inheritance [36]. However, since it is a rare disease with no official means of estimating incidence or prevalence, it is probably underreported.

## 4. Trehalose as a Low Glycemic-Index Sugar for Diabetes Mitigation

The over-consumption of added sugars has been associated with a heightened probability of many chronic diseases, including, diabetes, obesity, cardiovascular and liver diseases, cancer, and cognitive impairments [37]. Among these, diabetes is one of the most prevalent metabolic disorders, resulting in serious health complications [38]. Various treatments and medications have been formulated to modulate glucose homeostasis in diabetic individuals [39].

One of the proposed biological roles of trehalose is that it can potentially stabilize blood sugar levels through slower blood glucose release and a milder insulin response compared to other monosaccharides and disaccharides [40,41]. Oku et al. reported that in comparison to glucose, trehalose does not result in a rapid rise in blood sugar levels or a reduction in insulin secretion in female college students [42]. Yoshizane et al. found that 25 g trehalose (in 100 mL water) stimulated insulin and active incretin (a gut hormone released into the blood after eating that promotes insulin production) secretion less than glucose [41]. In contrast, when sucrose (composed of glucose and fructose) is consumed, it triggers a prompt increase in both blood glucose and insulin levels [43]. The findings of these studies indicate that, when compared to glucose ingestion, trehalose intake resulted in a diminished peak in blood glucose levels, not only suppressing the initial spike, but also producing a considerably reduced cumulative effect [44].

However, there are open questions remaining about the role of trehalose and its effects on blood glucose concentration. It is noteworthy that trehalose and glucose are metabolized differently in the human body, as the glucose released from trehalose is taken up by different parts of the gastrointestinal tract than that of trehalose itself. It was suggested that trehalose may impede glucose transport via the solute carrier 2A (SLC2A) transporter, which may explain the decrease in blood glucose levels [45]. However, once the brush border trehalase transforms trehalose into glucose, these glucose molecules can then be taken up by the uninhibited SLC2A transporters in the small intestine. Thus, it is possible that not every trehalose molecule gets broken down into glucose and taken up, and an unknown quantity of undecomposed trehalose can be found throughout the bloodstream after ingestion. These intact trehalose molecules may have physiological activities independent of providing two glucose molecules upon hydrolysis.

The molecular effects associated with trehalose modulating glucose homeostasis have been discussed and summarized in detail in a recent review [40]. The evidence points to trehalose consumption having a beneficial effect on hyperglycemic conditions by alleviating underlying pathological processes, through (1) enhancing insulin sensitivity by modulating the glucose signaling pathway, (2) reducing insulin secretion by reducing fat cell buildup, which is triggered by consuming carbohydrates and requires insulin to be processed, (3) normalizing glucose metabolism by modulating postprandial glucose levels, (4) modulating lipid metabolism by regulating postprandial insulin secretion, (5) enhancing pancreatic islet function by improving pancreatic beta cell function and preventing apoptotic processes associated with beta cell malfunction, (6) attenuating oxidative stress, which reduces free radical overload and improves insulin resistance, and (7) inhibiting inflammatory responses by ameliorating inflammatory mediators [40].

Taken together, these studies point to trehalose as a promising non-pharmaceutical agent for the control of glycemia in diabetic patients. More clinical trials, however, are needed to better characterize the effect of trehalose in mitigating diabetes symptoms. Further work to characterize the associated mechanism can be used to corroborate clinical effects, and potentially pave the way for the discovery of novel therapeutic avenues for other metabolic diseases.

## 5. Trehalose Effects on the Gut Microbiome

### 5.1. Alterations in Gut Microbiome in Response to Trehalose and Other Sugars

Changes in diet can have a variety of effects on the gut microbiome. Hundreds of species coexist in the human gastrointestinal tract, each thriving within a niche habitat determined by environmental factors, including nutrient availability, predators, and competitors [46]. It has been demonstrated that microbial composition can shift rapidly following a diet change [47]. A change in the amount of available nutrients in the environment can cause evolutionary pressure to be exerted on the whole gut microbial population [48]. New or existing mutations that improve nutrient sensing, transport, or metabolism and provide an advantage to their host are more favored. As a result, the microbiota is constantly adapting to changes in the availability of various sugars and other dietary components.

Trehalose and other dietary sugars or sweeteners impact the microbial populations that comprise the gut microbiome [49]. In the U.S., roughly half of all calories consumed come from carbohydrates, of which 13% derive from added sugars [50]. Simple sugars (e.g., glucose, fructose, high-fructose corn syrup, sucrose, and trehalose), sugar alcohols (e.g., sorbitol, erythritol, xylitol, and mannitol), and synthetic sugars (e.g., stevia, aspartame, saccharin, sucralose, and acesulfame potassium) are examples of commonly added sugars and sweeteners in modern Western diets [51]. In the past decades, synthetic sweeteners have been formulated and various natural sugars have been incorporated into food products [52]. The introduction of these sugars and sugar substitutes has prompted evaluations of how they affect the human body through changes in the composition and function of the gut microbiota. Indeed, multiple studies have demonstrated that microorganisms can swiftly alter their metabolism when exposed to a novel sugar [53,54]. A recent review highlighted three major strategies the gut microbiome can utilize to adjust to an increased consumption of sugar or sweeteners, including transcriptional changes, population compositional changes, and genetic changes [49].

First, microorganisms can adjust their transcription and metabolic activity in various ways, to adapt to different conditions in their environment, using regulatory signaling pathways, such as catabolite repression [49]. One example is that bacteria often modify the transcription and translation levels of key metabolic and transport proteins as the nutrient pool changes. The catabolite repression of glucose and fructose in *Bacteroides thetaiotaomicron* leads to the suppression of polysaccharide utilization genes, thus hindering its ability to colonize mouse gastrointestinal tracts [53]. Second, shifts in the microbiome composition can occur, allowing the microbes that are best suited to a given environment to become more prevalent. Research on trehalose as a prebiotic found that it significantly stimulated the growth of bacteriocin-producing lactic acid bacteria, particularly *Lactococcus lactis* spp. and *Lactococcus* sp. [55]. An investigation of the influence of a diet high in glucose or fructose on gut microbiota and intestinal permeability showed that these two highly consumed dietary sugars induce changes in the mouse microbiota, leading to a decrease in *Bacteroides* diversity and abundance, with a concurrent increase in *Proteobacteria* abundance [56]. Consumption of the artificial sweeteners sucralose and saccharin may also cause an imbalance in the microbiota composition [57]. The blood glucose regulation of a group of non-diabetic human participants was shown to be inferior when consuming saccharin, as indicated by their raised blood glucose levels [58]. Third, microorganism populations within the gut are able to modify their genetic makeup in response to changes in their environment, enabling them to make the most of new microhabitats, and allowing them to take advantage of new food sources. For example, the addition of galactitol, a sugar alcohol derived from galactose, caused the emergence and co-existence of a bacterial strain able to metabolize the galactitol alongside the original bacterial population [59]. It was found that these galactitol-positive strains were capable of using a rarely tapped galactitol ecosystem while coexisting with the galactitol-negative strains, which competed for alternative carbon sources with the collective microbiome.

There are many unexplored routes which have yet to be investigated regarding how microbes interact with sugars and sweeteners, and their effect on the host. Sugar alcohols, for example, were found to promote multiple beneficial microbes, although it is unclear whether this occurs through simple growth stimulation or more intricate connections [60]. It is critical to recognize that any change in the sugar and sweetener profile we consume redefines the nutrient environment available to our gut microflora. Further research into how trehalose, among other types of sugars and sweeteners, is able to affect the gut microflora population, activity, and effect on human physiology will continue to shed light on the interactions between diet, nutrition, and health.

### 5.2. Trehalose and Clostridioides difficile

A number of recent studies on the role of trehalose in microbiome modification has been focused on the pathogen *C. difficile*. It was first reported in 2018 that hypervirulent strains of *C. difficile*, a spore-forming Gram-positive bacterium, have acquired novel mechanisms for trehalose utilization, leading to an enhanced virulence and a more severe intestinal infection [61]. The two variant *C. difficile* strains, ribotypes RT027 and RT078, exhibited a noticeably higher efficiency in trehalose uptake, allowing for better growth in low trehalose concentrations. It was speculated that the rise in trehalose consumption, which was prompted by the approval of trehalose as Generally Recognized as Safe (GRAS) in 2000, may have contributed to the proliferation of these strains in humans [61].

All of the examined strains of *C. difficile* harbor trehalase TreA, a phosphotrehalase enzyme that is regulated by an upstream transcriptional regulator TreR [61]. TreA is important for *C. difficile* to grow on trehalose-containing media, as it catalyzes the transformation of trehalose-6-phosphate into glucose and glucose-6-phosphate. In RT027 isolates, sequence analysis implicated a single nucleotide polymorphism (SNP) within the *treR* gene causing de-repression of *treA*, allowing RT027 to activate the *treA* gene at 500-fold lower trehalose concentrations, explaining the increased trehalose utilization efficiency [23]. In the RT078 lineage, enhanced trehalose utilization is achieved through an alternative mechanism. In all RT078 strains that have been sequenced thus far, a four-gene addition was discovered, which is believed to encode a second copy of a phosphotrehalase (TreA2, with 55% amino acid identity to TreA), a potential trehalose-specific transport protein (PtsT), a putative glycan debranching enzyme (TreX) from the trehalase family, and a second copy of the TreR repressor (TreR2, with 44% amino acid identity to TreR) [62]. Mutational analysis of the transporter gene *ptsT* in RT078 was found to confer improved growth when trehalose levels were low [61]. Thus, increased trehalose consumption, combined with enhanced utilization of trehalose in these *C. difficile* epidemic strains, was suggested as potentially playing a causative role in the development of these hypervirulent ribotypes.

The potential of hypervirulent *C. difficile* strains to utilize dietary trehalose as a carbon source has led to the development of trehalase-resistant trehalose analogues as a more advanced metabolic treatment, which can retain the sweetener and potentially metabolic advantages of trehalose, and reduce the potential risks associated with its enzymatic breakdown. Two trehalose analogues, lactotrehalose and 5-thiotrehalose, have been discovered to resist enzymatic degradation, thus not able to serve as a carbon source for *C. difficile* [63]. Lactotrehalose inhibition was ribotype-dependent, as increasing lactotrehalose concentrations decreased growth of RT027, whereas RT078 was unaffected. The trehalose analog 5-thiotrehalose, on the other hand, can inhibit the growth of both RT027 and RT078 strains while remaining non-toxic to mammalian cells [63]. Thus, research into trehalose analogues that are resistant to degradation could be beneficial for their use as trehalase inhibitors, and as alternatives, or combined with trehalose in situations where controlling microbial growth and disease are important.

However, despite the suggestion that increasing trehalose consumption is related to increasing incidence of *C. difficile* infections, multiple subsequent studies have indicated this correlation is unlikely to be causative. In one study, a human gut model, which has been previously validated, was used to assess how trehalose influences the composition of human microbiota and the emergence of *C. difficile* infection (CDI) [64]. The researchers discovered that the human microbiome adapted swiftly to process all of the available trehalose, and when antibiotics altered the microbiota, trehalose supplementation did not cause CDI in comparison to glucose or saline supplementation [30]. A different study examining the impact of trehalose and lactotrehalose on the microbiome and severity of CDI reported that oral administration of trehalose reduced *C. difficile* abundance [65]. Eyre et al. investigated the prevalence of mutations influencing trehalose metabolism among CDI patients and the correlation of these mutations to the severity of the illness by analyzing the genetic diversity of *C. difficile* strains which had been previously sequenced [66]. The population was found to have a high prevalence of trehalose utilization variants, and the administration of trehalose did not generate greater concentrations of *C. difficile* or its spores than those that were seen with glucose or saline supplementation. On the contrary, trehalose administration lowered toxin detection to undetectable levels, though the cause was not clear [66]. Similarly, Saund et al. reported no correlation between trehalose utilization variants and severe cases of CDI among hospitalized patients, as demonstrated by the clinical data from 1144 CDI patient samples [62]. Both studies suggested that the presence of trehalose variants was far more extensive than previously anticipated, reflecting that the ability to utilize low concentrations of trehalose was acquired before the recent surge in trehalose usage. However, one limitation of these studies is that the majority of the examined isolates originated from the U.S. or Europe, despite the fact that trehalose consumption is higher in Asia. While further study could be useful to evaluate these geographic differences, a meta-analysis of 51 studies reported that CDI occurrence in Asia is comparable to that in Europe and North America [67]. Taken together, available evidence indicates trehalose consumption is not linked to increased *C. difficile* infections or the prevalence of strain variants more able to consume trehalose among *C. difficile* infections. However, this example highlights the utility of performing well-controlled experiments to evaluate the consequences of trehalose and other sugars or sweeteners on the gut microflora. Such studies have potential for the development of beneficial prebiotic supplements to promote healthy gut microflora.

## 6. Summary and Future Prospects

Trehalose is a naturally occurring, non-reducing sugar found in abundance in nature and has been used in many everyday products. For the past several decades, research has suggested that it may be a viable therapeutic agent to activate autophagy under several conditions in which autophagy is vital, such as neurodegenerative diseases, cancer, aging, cardiometabolic disorders, and infectious diseases [14,68,69,70,71]. It is speculated that the protective properties of trehalose are from autophagy induction and clearance of protein aggregates, however, these observations require further confirmation and validation in a clinical setting.

Trehalose has also been proposed as a non-pharmacological agent to regulate glucose levels in diabetic individuals. Studies have indicated that it could be effective in modulating glucose metabolism and maintaining glucose homeostasis by ameliorating inflammation and apoptosis, reducing postprandial insulin secretion, improving beta cell function, and normalizing the lipid profile [40]. This evidence suggests that trehalose could be a viable alternative to drug therapy for controlling blood sugar in diabetic individuals, however, further clinical trials are also required to validate this hypothesis.

In addition to potential therapeutic benefits, trehalose incorporated into diets can alter the composition of the human intestinal microbiota. Despite a lack of comprehensive research on the effects of trehalose on microbial populations, the introduction of novel sugars and sweeteners can drastically remodel the gut habitat, which in turn affects microbial metabolism and metabolite secretion [49]. One illustration is the recently proposed association between a *C. difficile* outbreak and increased trehalose dietary consumption, though more recent research presented contradictory results and indicated no direct correlation between trehalose consumption and *C. difficile* infections [30,72]. However, this raised the general concern that pathogens may use trehalose or other rare sugars to gain an advantage in the gut microflora, which has led to developing trehalase-resistant trehalose analogues. These molecules may help retain or enhance the metabolic benefits of trehalose, and mitigate the potential risks of microbial drug metabolism.

Taken together, the studies examining trehalose as a bioactive nutrient have yielded interesting insights into its potential to treat various human diseases, as well as its importance in the gut microbiota. It is likely that trehalose has multiple, distinct effects that contribute to its ability to reduce the severity of metabolic diseases. Further work remains to be performed to elucidate the complete scope of the biological impacts associated with trehalose.
